# Bradykinin Receptor B1 and C-Reactive Protein as Prognostic Factors for Pharyngocutaneous Fistula Development After Laryngectomy

**DOI:** 10.1007/s12105-019-01043-z

**Published:** 2019-06-27

**Authors:** Isabelle Koob, Anja Pickhard, Maria Buchberger, Melanie Boxberg, Rudolf Reiter, Guido Piontek, Ulrich Straßen

**Affiliations:** 1 Department for Ear- Nose- and Throat, Head and Neck Surgery, University Hospital Klinikum rechts der Isar, Technical University of Munich, Ismaninger Str. 22, 81675 Munich, Germany; 2 Institute of Pathology, Technical University of Munich, Trogerstraße 18, 81675 Munich, Germany; 3 Department of Otolaryngology Head and Neck Surgery, Section of Phoniatrics and Pedaudiology, University of Ulm, Ulm, Germany

**Keywords:** Vascular endothelial growth factor receptor, Bradykinin receptor, Pharyngocutaneous fistula, Laryngectomy, Inflammation

## Abstract

Pharyngocutaneous fistulae (PCF) are one of the most common complications after laryngectomy. Predisposing risk factors have been studied, yet knowledge to determine which patients are prone to developing a fistula remains scarce. This study aims to establish prognostic parameters to identify individual patients at risk for PCF development. As PCF and inflammation seem to be interwoven, this work focuses on markers able to detect an inflammatory response. We retrospectively analyzed all patients who had undergone a laryngectomy at our clinic in the years 2007 to 2017 (n = 182). Immunohistochemical expression of bradykinin type 1 and 2 receptor and vascular endothelial growth factor receptor 2 was studied in all available tumor samples. Additionally, the clinical inflammation parameters ‘body temperature’, ‘pain’, ‘c-reactive protein (CRP)’, and ‘leucocytes’ were postoperatively tracked in all patients. The times between fistula diagnosis, therapeutic approach, and hospital discharge were recorded. We found a strong correlation between inflammation and the formation of a fistula. High bradykinin 1 receptor expression in the tumor samples correlated with postoperative PCF development. Persistently elevated CRP and leukocyte levels beyond the 6th postoperative day were also risk factors. A decreased time lapse between PCF diagnosis and surgical revision clearly correlated with a shorter hospital stay. In this study, we identified a bradykinin 1 receptor positive patient group at high risk for development of PCF. We recommend close monitoring for fistula formation in these patients to ensure timely intervention.

## Introduction

Pharyngocutaneous fistula (PCF) is one of the most common early postoperative complications after total laryngectomy. The reported incidence of PCF varies widely and ranges from 2.6 to 65.5% depending on the selected patient cohort [1, 2]. On average, the incidence of PCF after head and neck surgery is around 20%.

PCF is caused by a disruption of the pharyngeal mucosal suture which results in a communication between the neopharynx and cervical skin. Permanent salivary leakage from the communication leads to ongoing inflammation that inhibits proper wound healing and the onset of complementary therapy. This prolongs hospitalization, increases the costs of treatment, and, most importantly, strains the patient’s physical and psychological well-being [3–5].

Many studies have focused on identifying potential risk factors for PCF. Previous radiotherapy and/or chemoradiotherapy, tumor origin, and diabetes are established factors associated with PCF [6–8]. Knowledge of predisposing factors contributing to PCF formation is established, but details on reliable and timely identification of individuals developing a postoperative PCF remain insufficient. Early prediction of PCF formation would allow for prompt imaging diagnostics and subsequent revision surgery. Studies have indeed displayed the beneficial effect of early surgical intervention on morbidity and duration of hospitalization in PCF patients [9, 10].

In this study, the main goal was to trace parameters of ongoing formation of a PCF in patients after laryngectomy. As PCF and inflammation are closely linked, the focus was on indicators of an inflammatory response. Besides the known risk factors previously mentioned, rarely evaluated factors, such as clinical and morphologic inflammation indicators, were analyzed for predictive power for PCF formation. Parameters easily assessable during clinical routine were chosen to include body temperature, pain, C-reactive protein (CRP) and leucocytes.

Tissue markers capable of detecting inflammation in the tumor and its surrounding environment were also evaluated. These included bradykinin 1 receptor (B1-R), bradykinin 2 receptor (B2-R), and vascular endothelial growth factor receptor 2 (VEGF-R2). Bradykinin is part of the kinin-kallikrein family and important to the induction of an inflammatory response. It acts via inducible B1- and constitutive B2-receptors in injured tissue and may constitute a promising prognostic indicator for PCF formation. The formation of blood vessels is also necessary for the maintenance and progression of inflammation. As an important protein in vasculogenesis, VEGF-R2 was additionally evaluated as a potential prognostic indicator for PCF.

By defining reliable and early parameters of PCF-formation, we aimed to shorten the time to PCF diagnosis, expedite appropriate treatment, and reduce convalescence.

## Materials and Methods

### Patient Cohort

The patient cohort of this retrospective study consisted of all patients who had undergone laryngectomy (regardless of the surgery indication) at our clinic between January 2007 and January 2017 (n = 182). Detailed patient characteristics are presented in Table 1.

**Table 1:** Overview of patient cohort

Patient collective	
Overview	182 (160 male/22 female)
Average age	63 [95% confidence interval: 62–65]
PCF^†^/no PCF^†^	45 (25%)/137 (75%)

TNM	TNM-Status	Total (n^‡^)	PCF (%)	No PCF (%)
T-status	T1	8	1 (12.4)	7 (87.5)
	T2	45	17 (37.8)	28 (62.2)
	T3	52	11 (21.2)	41 (78.8)
	T4	69	15 (21.7)	54 (78.3)
	Total	174	44 (25.3)	130 (74.7)
N-status	N0	84	26 (31)	58 (69)
	N1	25	5 (20)	20 (80)
	N2	63	13 (20.6)	50 (79.4)
	N3	2	0 (0)	2 (100)
	Total	174	44 (25.3)	130 (74.7)
M-status	M0	169 (97%)	43 (25.4)	126 (74.6)
	M1	5 (3%)	1 (20)	4 (80)
	Total	174 (100%)	44 (25.3)	130 (74.7)
Grading	G0	1	1 (100)	0 (0)
	G1	7	4 (57.1)	3 (42.9)
	G2	97	21 (21.6)	76 78.4)
	G3	63	16 (25.4)	47 (74.6)
	Total	168	42 (25)	126 (75)

Main tumor	Tumour entity	Total (n^‡^)	PCF (%)	No PCF (%)
Hypopharynx	SCC*	55	19 (34.5)	36 (65.5)
Larynx	SCC*	111	23 (20.7)	88 (79.3)
Hypopharynx and Larynx	SCC*	4	1 (25)	3 (75)
Thyroid gland	Papillary	2	0 (0)	2 (100)
	Follicular	2	1 (50)	1 (50)
	Total	4	1 (25)	3 (75)
Synovial sarcoma		2	0 (0)	2 (100)
non tumorous entities	Alkali burns	2	1 (50)	1 (50)
	Laryngeal stenosis	1	0 (0)	1 (100)
	Radiogenic necrosis	1	0 (0)	1 (100)
	No specification	2	0 (0)	2 (100)

Risk factors	n Analysed	n^‡^ Applicable	PCF (%)	Non PCF (%)
Alcohol	166	119	31 (26)	88 (74)
Nicotine	166	119	33 (27.7)	86 (72.3)
Diabetes	167	31	8 (25.8)	23(74.2)
Previous RT^§^	182	20	4 (20)	16 (80)
Previous RCT^§^	182	16	10 (62.5)	6 (37.5)
Pectoralis Flap	182	3	1 (33.3)	2 (66.7)
Radialis Flap	182	18	5 (27.8)	13 (72.2)

^§^*RT* radiotherapy; *RCT* radiochemotherapy

^†^*PCF* pharyngocutaneous fistula

^‡^ Number of valid cases; Percentage indication refers to number of valid cases

*squamous cell carcinoma

All risk factors for PCF described in the literature and available for retrospective chart analysis were recorded (Table 1). In addition to the acknowledged risk factors, surgeons performing the laryngectomy were recorded in an anonymized manner.

Inflammation parameters were investigated with regard to their diagnostic power. In order to obtain a broad diagnostic spectrum, preoperative morphologic and postoperative clinical parameters were subject to analysis. The former comprise the protein markers B1-R and B2-R as well as VEGF-R2. The latter include the ten-day postoperative course of temperature and pain (VAS) and 15-day postoperative survey of serum inflammation values CRP and leucocytes.

### Immunhistochemical Staining

The protein marker expression was analyzed using tumor tissue sections and non-cancerous mucosa from the resection margins of all available histopathologic specimens (n = 156) (Table 2). Specimens without tumor were excluded from immunohistochemical analysis. Fresh 1.5 µm sections from formalin-fixed and paraffin-embedded samples were transferred to glass slides, dewaxed, and rehydrated. Antigen retrieval (microwave oven heating in citrate buffered saline for B1-R and B2-R and in EDTA buffered saline for VEGF-R2) was performed according to the manufacturers’ recommendations. After cooling, the slides were incubated with the primary antibody (Table 3, additional files). The reaction was developed with the labeled streptavidin–biotin-alkaline phosphatase system using DAB as a reaction indicator. After counterstaining with hematoxylin, the slides were dehydrated using ascending ethanol concentrations and mounted. Tissue samples known to express the respective antigens were used as a positive control. Antibodies of irrelevant specificity with an immunoglobulin isotype identical to that of the primary antibody were used as negative controls.

**Table 2:** Overview of patient cohort of the immunohistochemical analysis

Patient collective of the immunohistochemical analysis	
Overview	156 [150 male/16 female]
Age	64 [95% confidence interval: 62–65]

	B1-R			B2-R			VEGF-R2		
PCF^†^	PCF^†^	No PCF^†^	n^‡^	PCF^†^	No PCF^†^	n^‡^	PCF^†^	No PCF^†^	n^‡^
	33(23%)	113(77%)	146	37(25%)	113(75%)	150	37(26%)	106(74%)	143

Risk factor	Applicable (%)	Not applicable (%)	n^‡^	Applicable (%)	Not applicable (%)	n^‡^	Applicable (%)	Not applicable (%)	n^‡^
Alcohol	37 (27)	99 (73)	136	38 (25)	102 (75)	140	36 (27)	97 (73)	133
Nicotine	75 (55)	62 (45)	137	77 (55)	64 (45)	141	73 (54)	61 (46)	134
Diabetes	28 (20)	111 (80)	139	29 (21)	112 (79)	141	27 (20)	107 (80)	134
Previous RT	27 (18)	119 (82)	146	26 (17)	124 (83)	150	26 (18)	117 (82)	143
Previous RCT	13 (9)	133 (91)	146	13 (9)	137 (91)	150	13 (9)	130 (91)	143

TNM	TNM-satus	n	%	TNM-satus	n	%	TNM-satus	n	%
T-status	n^‡^ = 145 (100%)			n^‡^ = 149 (100%)			n^‡^ = 142 (100%)		
	T1	7	5	T1	7	5	T1	7	5
	T2	37	25	T2	39	27	T2	35	25
	T3	43	30	T3	45	39	T3	42	29
	T4	58	40	T4	58	39	T4	58	41
N-status	n^‡^ = 145 (100%)			n^‡^ = 149 (100%)			n^‡^ = 142 (100%)		
	N0	74	51	N0	72	48	N0	70	49.5
	N1	21	15	N1	23	16	N1	23	16
	N2	48	33	N2	52	35	N2	47	33
	N3	2	1	N3	2	1	N3	2	1.5
M-status	n^‡^ = 143 (100%)			n^‡^ = 146 (100%)			n^‡^ = 139 (100%)		
	M0	138	97	M0	141	97	M0	134	96
	M1	5	3	M1	5	3	M1	5	4

^†^*PCF* pharyngocutaneous fistula

^‡^Number of valid cases; Percentage indication refers to number of valid cases

**Table 3:** Additional files: Characteristics of antibodies used for immunohistochemical staining

Antibodies	Dilusion	Manufacturer
Rabbit Anti-Bradykinin Receptor Type B1	1:100	Thermo Fisher Waltham. MA, USA
Rabbit Anti-Bradykinin Receptor Type B2	1:100	US Biological Salem. MA, USA
Rabbit Anti-VEGF Receptor 2 antibody	1:100	Abcam Cambridge. UK

**Fig. 1:**
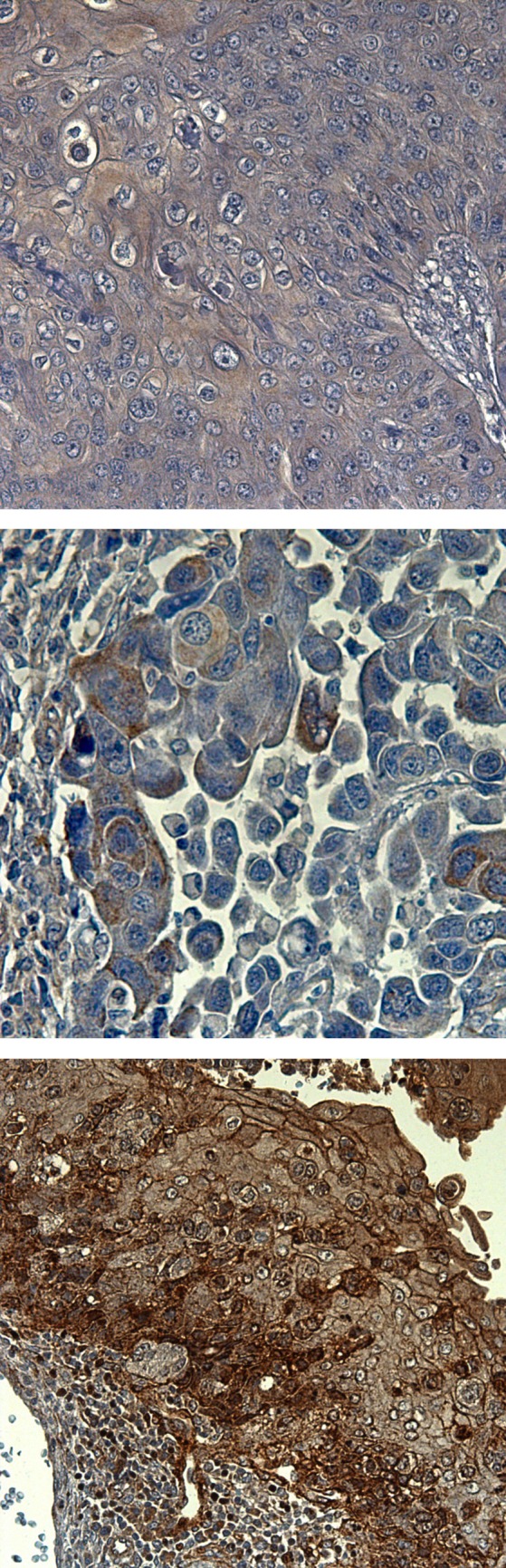
Exemplary demonstration of varied immunohistochemical staining scores for B1-R. from top to bottom: B1-R immunoreactive score negative; B1-R immunoreactive score = 2; B1-R immunoreactive score = 5

**Fig. 2:**
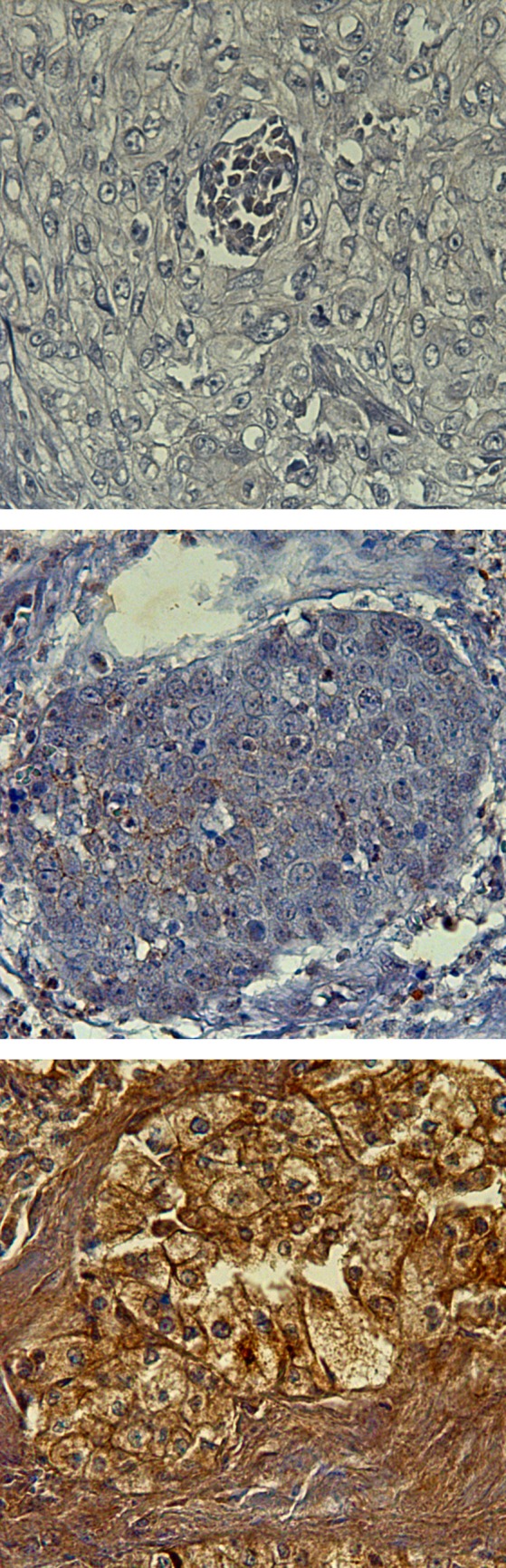
Exemplary demonstration of varied immunohistochemical staining scores for B2-R. from top to bottom: B2-R immunoreactive score negative; B2-R immunoreactive score = 2; B2-R immunoreactive score = 5

The outcome of the antibody-expression was graded by the immunoreactive score displayed in Table 4 in the additional files. A graphic illustration of the respective scores is conveyed in Fig. 1, 2, 3. For B1-R and B2-R, the membrane-bound and cytoplasmic expression was analyzed. For VEGF-R2, membrane-bound expression was distinguished from endothelial vessel expression. The latter was evaluated by means of quantitative categorization of vascular density. In order to assess vascular density, immunohistochemical tissue samples were examined through a 10 × objective and the vessels visible in every field of vision (fov) were counted. Fewer than five vessels in every fov was categorized as low expression, whereas five and more vessels in at least one fov was categorized as high expression.

**Table 4:** Additional files: Immunoreactive score

Membrane-bound immunoreactive score = PP^†^ + SI^‡^				Cytoplasmatic score	
PP^†^		SI^‡^		SI^‡^	
negative	0	negative	0	negative	0
< 10%	1	weakly positive	1	weakly positive	1
10–29%	2	moderately positive	2	strongly positive	2
30–60%	3	strongly positive	3	/	3
> 60%	4	/	4	/	4

^†^*PP* percentage points: percentage of stained tumor cells

^‡^*SI* staining intensity

**Fig. 3:**
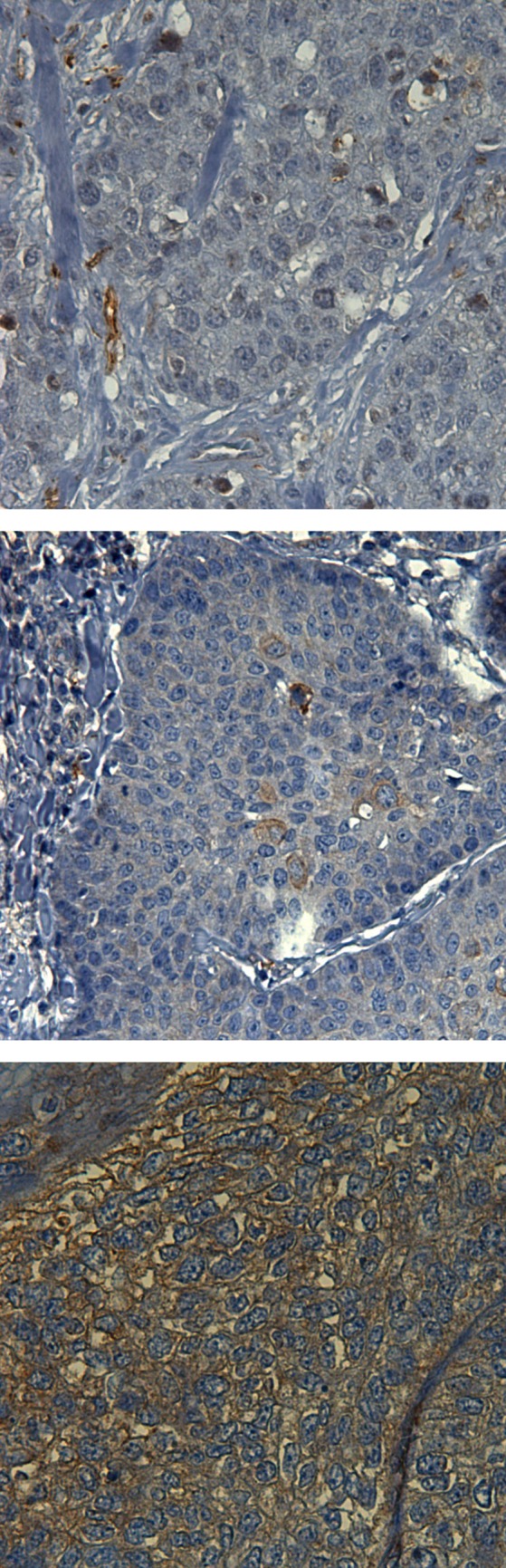
Exemplary demonstration of varied immunohistochemical staining scores for VEGF-R2. From top to bottom: VEGF-R2 immunoreactive score negative; VEGF-R2 immunoreactive score = 2; VEGF-R2 immunoreactive score = 5

### Statistics

Statistical analyses were conducted using IBM^®^ SPSS^®^ Statistics 24. Descriptive statistics were used to characterize the variables in the patient population. Continuous variables were analyzed by *t* test or Mann–Whitney rank sum test, and categorical variables were compared using the Chi squared test or, alternatively, Fisher’s exact test. A p-value of < 0.05 indicated statistical significance.

Risk factors were analyzed by means of the Chi square or Fisher’s exact test respectively.

The relative risk and its 95% confidence interval were calculated by means of the fourfold Chi squared test. Individual parameters showing a significant association with PCF development in the univariate model were analyzed in a logistic regression model (binary logistic regression approach using log-rank) in order to determine their power of affecting the probability of PCF formation.

Receiver operating characteristic curves and Youden-index were used to ascertain ideal cut-off values for CRP and leucocytes.

Surgeons’ PCF-rate was calculated and adjusted for the date of the surgery, thus reflecting surgeons’ evolution of experience. Ultimately, Pearson’s correlation was computed between the amount of laryngectomies performed and surgeons’ adjusted PCF-rate.

## Results

### Elevated CRP and Leukocyte rates in Blood Samples Reveal a Fistula

**Fig. 4:**
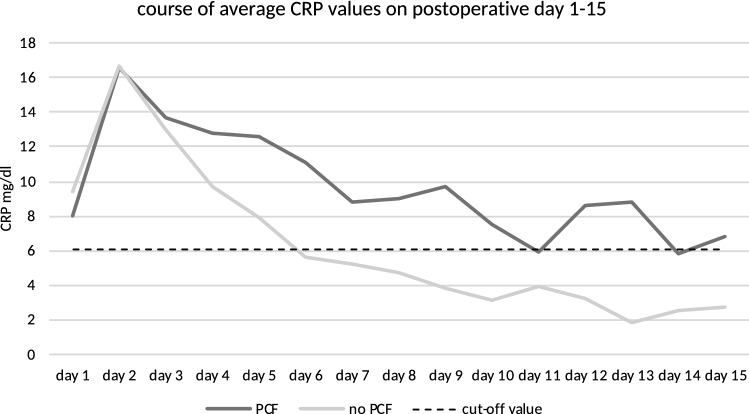
Development of postoperative average CRP values in relation to the determined cut-off value of 6.1 mg/dl

**Fig. 5:**
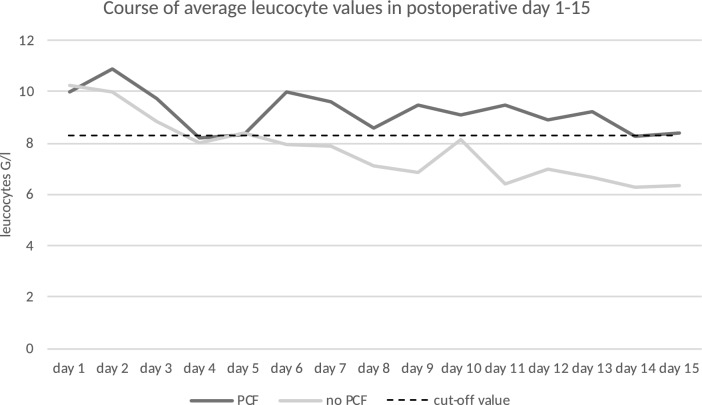
Development of postoperative average leucocyte values in relation to the determined cut-off value of 8.3 G/l

**Fig. 6:**
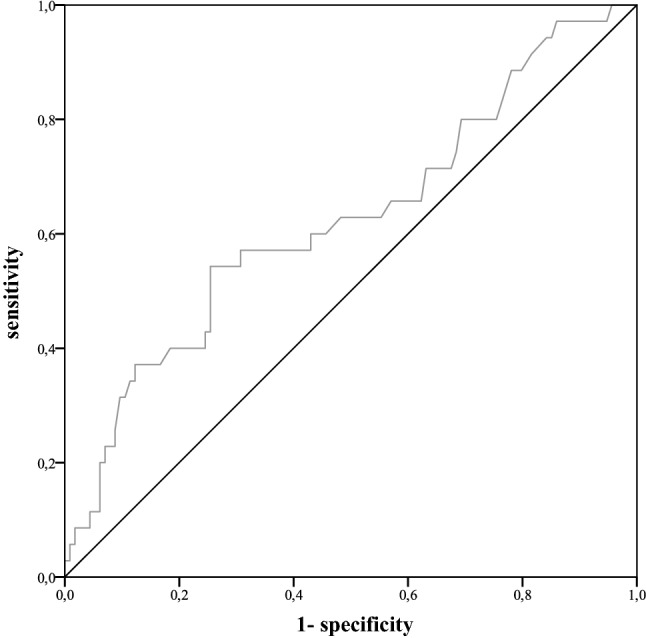
ROC curve for CRP values on postoperative day 6–15

**Fig. 7:**
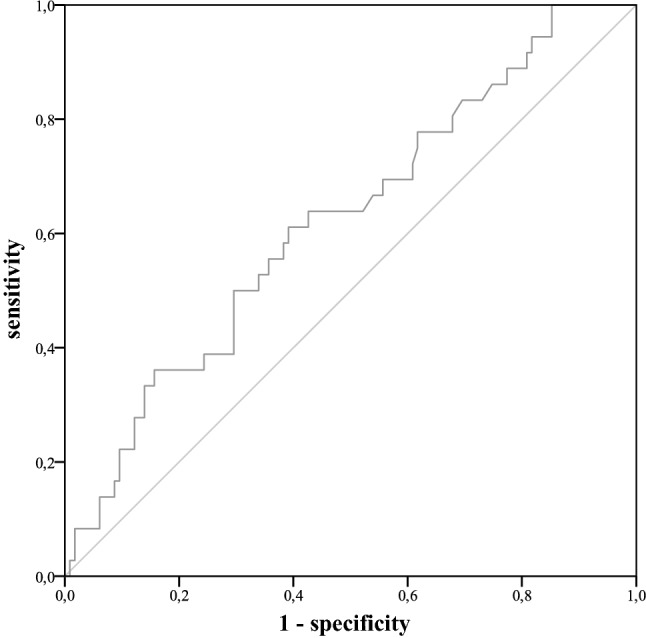
ROC curve for leucocyte values on postoperative day 6–15

The median day of PCF diagnosis was postoperative day 11 (range 3–28). Twenty-five percent (n = 45) of the included patients developed PCF. These patients showed a significantly (p < 0.001) longer hospital stay after laryngectomy (mean 59 days) than patients without PCF (mean 26 days). PCF-patients also showed significantly higher average values of CRP and leucocytes during the postoperative period (p_CRP_ = 0.024, p_leucocytes_ = 0.026). For the remaining clinical parameters, pain and body temperature, no significant differences were established. As CRP and leucocytes started to differ around the 6th postoperative day between PCF-patients and non-PCF-patients, cut-off values were determined for days 6–15 (Fig. 4 and Fig. 5). Optimal cut-off values were determined to be 6.1 mg/dl for CRP and 8.3G/l for leucocytes (Fig. 6 and Fig. 7). With the set cut-off-values combined, a sensitivity of 0.43, specificity of 0.84, positive predictive value of 0.45, and negative predictive value of 0.83 were achieved. If both leucocytes and CRP exceeded the cut-off values, the relative risk for fistula development was 2.63.

### Previous Radiochemotherapy and Surgical Skills are Independent Predictors for PCF-Formation

Out of the analyzed preoperative risk factors, only two proved to be of significant statistical relevance in our cohort: PCF is associated with previous radiochemotherapy and with the specific surgeon performing the laryngectomy. The logistic regression analyses confirmed that the factors ‘previous radiochemotherapy’ (odds ratio = 57.490) and ‘surgeon’ (odds ratio = 14.925) are independent predictors for PCF-formation. A detailed overview of all the analyzed risk factors and their relative risk is presented in Table 5.

**Table 5:** Evaluation of risk factors via Chi squared test (Fisher’s exact test when applicable) and calculation of relative risks

Risk factors	n^‡^ total risk factor (PCF and No-PCF)	n^‡^ risk factor PCF^†^	Significance	Relative risk for PCF
Overview patient collecitve	182 (100%)	45 (25%)		
Toxicants	${\text{n}}^{\ddag }$_alcohol and nicotine_ = 166 ${\text{n}}^{\ddag }$_diabetes_ = 167	${\text{n}}^{\ddag }$_nicotine and diabetes_ = 44 ${\text{n}}^{\ddag }$_alcohol_ = 43		
Alcohol	119 (72%)	31 (72%)	p = 0.945	1.020
Nicotine	119 (72%)	33 (75%)	p = 0.569	1.185
Diabetes	31 (19%)	8 (20%)	p = 0.857	1.064
Main tumor	n^‡^ = 174	n^‡^ = 44	p = 1.12	
Hypopharynx	55 (33%)	19 (46%)	p = 0.054	1.667
Larynx *(reference)*	111 (67%)	22 (54%)		
Hypopharynx & larynx	4 (3.5%)	1 (4.2%)	p = 1,00^eF^	1.207
Thyroid gland	4 (3.5%)	1 (4.2%)	p = 1.00^eF^	1.207
Further entities	8 (6.7%)	1 (4.2%)	p = 1.00^eF^	0.603
TNM	n^‡^ = 174	n^‡^ = 44		
T-status				
T1	9 (5%)	1 (2%)	p = 0.244^eF^	0.294
T2 (reference)	45 (26%)	17 (39%)		
T3	51 (29%)	11 (25%)	p = 0.072	0.560
T4	69 (39%)	15 (34%)	p = 0.062	0.575
N-status				
N0 (reference)	85(49%)	26 (59%)		
N1	25 (14%)	5 (11%)	p = 0.301	0.654
N2	63 (36%)	13 (29%)	p = 0.174	0.675
N3	2 (1%)	0 (0%)	p = 1.000^eF^	
M-status				
M0 (reference)	170 (97%)	43 (98%)		
M1	5 (3%)	1 (2%)	p = 1.000^eF^	0.791
RT & RCT^§^	n^‡^ = 182	n^‡^ = 45		
Previous RT^§^	20 (11%)	4 (9%)	p = 0.786	0.790
Previous RCT^§^	16 (9%)	10 (22%)	p = 0.001*	2.964*
Surgery related risk factors				
Voice prosthesis	150 (86%)	34 (77%)	p = 0.064	0.567
Surgeon			p = 0.031*	3.250*
Flap transplant	21 (12%)	6 (13%)	p = 0.664	1.179
Radialis-flap	18 (10%)	5 (11%)	p = 0.775^eF^	1.147
Pectoralis-flap	3 (2%)	1 (2,5%)	p = 0.570^eF^	1.376
No transplant	161(88%)	39 (87%)		
Preoperative parameters				
Anaemia	54 (43%)	15 (44%)	p = 0.862	1.053
Hypothyroidism	5 (7%)	2 (12%)	p = 0.330	1.813
AST/ALT > 1	56 (64%)	18 (75%)	p = 0.201	1.661

Anaemia♂defined as haemoglobin < 13 g/dl for men and < 12 g/dl for women

Hypothyroidism defined as thyreoglobine stimulating hormone > 4.00 µU/ml

*Statistically significant

^†^*PCF* Pharyngocutaneous fistula

^§^*RT* radiotherapy; *RCT* radiochemotherapy

^‡^Number of valid cases; Percentage indication refers to number of valid cases

^eF^ Fisher’s exact test

Furthermore, a strong negative correlation (r = −0.832) was established between the number of performed laryngectomies and surgeons’ adjusted PCF-rate (p < 0.001).

None of the clinicopathologic variables showed significant group differences in the Chi squared test.

### B1-R and VEGF-R2 Expression are Independent Predictors for a Postoperative PCF Formation

Immunohistochemistry revealed that membrane-bound and cytoplasmic expression of all three morphological markers, B1-R, B2-R and VEGF-R2, was significantly stronger in the tumor samples as compared to the normal tissue (p < 0.001).

VEGF-R2 high endothelial-bound expression (≥ 5 vessels/field of vision) correlated with PCF development (p = 0.003). Also, B1-R significantly correlated with PCF formation for B1-R membrane-bound expression ≥ 5 (p = 0.036). The relationship between the different marker-scores as well as risk for PCF-formation for the respective score is displayed in Table 6.

**Table 6:** Chi squared tests (or exact Fisher’s test respectively) between PCF and morphological markers as well as PCF-risk for respective score cut-off (± CRP > 6.1 mg/dl)

Markers	Localisation in the cell	Score	Significance	PCF-risk for markers solely	PCF-risk for CRP ≥ 6.1 mg/dl & markers
B1-R	Membrane	≥ 2	0.906	0.219	0.375
		≥ 3	0.379^eF^	0.3	0.5
		≥ 4	0.117^eF^	0.444	1.000
		≥ 5	0.036*^eF^	0.75	1.000
	Cytoplasm	≥ 1	0.478	0.247	0.456
		= 2	0.176	0.333	0.333
B2-R	Membrane	≥ 2	0.112	0.375	0.6
		≥ 3	1.000^eF^	0.272	0.5
		≥ 4	0.337^eF^	0.000	0.000
		≥ 5	0.572^eF^	0.000	0.000
	Cytoplasm	≥ 1	0.849	0.258	0.529
		= 2	0.738	0.308	0.666
VEGF-R2	Membrane	≥ 2	0.869	0.268	0.5
		≥ 3	0.781^eF^	0.211	0.5
		≥ 4	0.339^eF^	0.000	0.000
		≥ 5	0.569^eF^	0.000	0.000
	Vessels	≥ 5/fov	0.003*	0.337	0.519

*Statistically significant

^eF^ Fisher’s exact test

Logistic regression analysis showed that both B1-R membrane expression ≥ 5 and high VEGF-R2 endothelial expression were independent predictors for postoperative PCF formation with odds ratios of 12.167 and 4.812 respectively.

### Combined Findings of CRP ≥ 6.05 mg/dl and B1-R or VEGF-R2 Correlates with a Postoperative PCF Development

Chi squared tests demonstrated significant correlations between PCF development and the combined finding of both a CRP-value > 6.05 mg/dl and either a B1-R score ≥ 4 (p = 0.039), a membrane-bound VEGF-R2 score ≥ 2 (p = 0.033), or a high endothelial expression of VEGF-R2 (p < 0.001). A postoperative leucocyte value > 8.3 G/l only showed a significant correlation with PCF development when combined with a high endothelial expression of VEGF-R2. In the case of a combined finding of a B1-R score ≥ 4 and a CRP value > 6.1 mg/dl, the risk to develop PCF was 100%.

## Discussion

PCF remains one of the most common and challenging complications after laryngectomy. A PCF may turn a short hospital stay into a prolonged ordeal complicated by complex wound care, delayed onset of voice rehabilitation and oral diet initiation, and accessory revision surgery [11]. To avoid, or at least minimize, this sequelae, it is imperative to identify high risk patients.

### Risk Factors

Many studies have investigated contributing risk factors to PCF with often controversial results [8, 12–15]. Moreover, the documented preoperative risk factors can only be influenced to a very limited extent.

This study identified only two independent risk factors out of the numerous acknowledged and analyzed factors: previous radio chemotherapy (p = 0.001) and the influence of the surgeon (p = 0.031). Interestingly, radiotherapy alone, though often reported as a predisposing factor for PCF formation due to tissue scarring, did not show a correlation with PCF [1, 6, 16–19]. Combined radiochemotherapy, however, seemed to significantly raise the risk for PCF development. With these results, our study is in agreement with many others that did not find an increased risk for PCF after radiotherapy alone [20–22].

The second risk factor significantly associated with PCF in our study was the surgeon performing the laryngectomy. Contrary to the risk factor ‘previous radiochemotherapy’, this a variable may be influenced at the time of surgery and, therefore, is of great value. The development of PCF may depend on the surgeon’s technique. This includes meticulous hemostasis, especially in the time and care dedicated to the pharyngeal suture. Additionally, the impact of different suture techniques utilized by surgeons in pharyngeal closure was analyzed in several studies [22–25]. For example, interrupted sutures were shown to have a detrimental effect on PCF formation in comparison to continuous sutures [24]. The results of our study furthermore delineate that a surgeon’s PCF-rate is associated with their experience. It is commonly accepted that surgical skills are honed through practice. Our results substantiate this with PCF formation decreasing with increased numbers of laryngectomies performed per surgeon.

In accordance with the literature, we also demonstrated that the insertion of a voice prosthesis during the initial procedure was not a significant risk factor for the development of PCF [19, 26–28].

### Clinical Parameters

As PCF development seems to be closely linked to inflammation, we focused on readily available clinical inflammation values. Postoperatively elevated temperature has been described as a relevant indicator for PCF in prior studies [3, 9, 13, 19, 26]. These results should be interpreted with caution, however, as they are mostly obtained during the early postoperative period when other causes for fever have to be taken into consideration. In our study, we could not find an association between postoperative fever and PCF formation. Nor could postoperative pain, measured by VAS, be established as an indicator for PCF development.

On the other hand, postoperative serum inflammation values (CRP and leucocytes) demonstrated promising potential as detectors for PCF formation. Previous studies on leucocytosis have been inconclusive. Schwartz et al. focused on preoperative leucocytosis, which could not be established as risk factor [16]. Mäkitie et al. found that leucocytosis on the first postoperative day predicted PCF formation [26]. It must be considered, however, that it is normal to find elevated inflammation values in the early postoperative phase due to the recent surgical trauma. In later postoperative healing stages the CRP and leucocytes should drop. In our study, persistently elevated values of CRP > 6.1 mg/dl and leucocytes > 8.3 G/l past postoperative day 6 indicated a maintained inflammatory response and the formation of PCF. A close postoperative tracking of CRP and leucocytes could be harnessed as a tool to identify individual patients prone to PCF development after laryngectomy. While useful as this method may be included in routine clinical practice, it should be noted that the sensitivity remains low (43%).

### B1-R and VEGF-R2

The association between PCF and serum inflammation markers indicate that fistula formation and inflammation are closely connected.

The chosen tissue markers in this study are all directly or indirectly involved in inflammatory processes. The two bradykinin-receptors, B1-R and B2-R, play a crucial role in the inflammatory reaction via pain mediation, vasodilatation, and edema, as well as smooth muscle contraction and relaxation [29–31]. A continuous tumoral inflammatory response also requires the formation of a vascular network to enable the migration of inflammatory cells. Neoangiogenesis was initially revealed through VEGF-R2 antibodies.

Overall, all the markers studied showed an over-expression in the tumor samples in contrast to normal tissue. This finding confirms an inflammatory process in cancerous tissue. Prior studies have already demonstrated the over-expression of B1-R in prostate carcinoma, B2-R in human gliomas, and VEGF-R2 in inflammatory breast cancer [32–34]. In HNSCC, however, solely B2R was identified as over-expressed [35, 36].

When evaluating over-expression by means of a score, it becomes clear that high scores of certain markers correlate with postoperative PCF formation. Interestingly, it is not the ubiquitously occurring B2-R that correlates with a subsequent PCF formation, but rather B1-R, whose expression is solely up-regulated in pathophysiological conditions. Also, a high vessel expression in the tumor sample, depicted by VEGF-R2, was associated with postoperative PCF development. These findings suggest that the inflammatory response is not triggered through PCF formation but that underlying inflammation present in the operating tumor field might favor postoperative PCF formation.

Furthermore, the morphological markers B1-R and VEGF-R2 appear to be closely linked to CRP > 6.1 mg/dl past the 6th postoperative day, with a significant association in the Chi squared test. Positive testing of both B1-R ≥ 4 and CRP > 6.1 mg/dl in a patient yields a PCF-rate of 100%.

### Management

PCF requires an encompassing management from prevention to treatment. An accurate preoperative assessment of risk factors, perioperative diligence, and postoperative vigilance for impending complications are essential. In the postoperative period, an early recognition of PCF formation is crucial in order to prevent secondary wound complications. Indeed, primary closure, not to mention conservative management, becomes problematic if the diagnosis is delayed. The chronic inflammation and supplementary infection leads to poor vascular conditions and, finally, necrosis. It is estimated that an early diagnosis and subsequent adequate treatment are essential to a successful recovery process in postoperative management of PCF [9, 11, 37]. The extent of a surgical revision depends on the fistula’s dimension and contamination.

The findings of our study suggest an algorithm to facilitate early PCF diagnosis and hence a prompt surgical intervention: Preoperatively, high risk patients should be identified by screening for existing risk factors as well as for the expression of a B1-R membrane-bound score ≥ 5 or a VEGF-R2 endothelial expression ≥ 5 vessels/fov in the tumor samples. For selected high risk patients, a prophylactic flap-reinforced closure during laryngectomy should be taken into consideration in order to minimize the odds for a later PCF development [37–40]. Postoperatively, CRP and leucocytes should be closely tracked in every patient but with special attention in high risk patients. Any elevation of CRP and leucocytes above the set cut-off values beyond postoperative day 6 should, with regard to the specificity (84%) of the test, be followed by prompt imaging diagnostics in order to exclude or confirm a PCF. A newly developed fistula should be treated by surgical intervention in a timely manner to decrease the patient’s length of hospitalization.

To our knowledge, we are the first to propose a diagnostic algorithm based on the expression of inflammatory parameters. Such a diagnostic tool could be of great value in both reducing the financial costs of PCF and, most importantly, assisting the patient’s physiological and psychological recovery.

Due to the retrospective nature of this current study, however, it is recommended that this algorithm be further supported by way of prospective research.

## Conclusions

Patients showing a B1-R membrane-bound score ≥ 5 or a VEGF-R2 endothelial score ≥ 5 vessel/fov should be categorized as high-risk patients for PCF development. Furthermore, a postoperative elevation of CRP ≥ 6.1 mg/dl or leucocytes ≥ 8.3 G/l beyond the 6th postoperative day, especially in high-risk patients, should be followed by immediate imaging diagnostics and surgical intervention. These measures will aid to avoid disease progression and thereby shorten the length of hospitalization.
